# First-Principle Study on p-n Control of PEDOT-Based Thermoelectric Materials by PTSA Doping

**DOI:** 10.3390/polym13203518

**Published:** 2021-10-13

**Authors:** Hideki Arimatsu, Yuki Osada, Ryo Takagi, Takuya Fujima

**Affiliations:** Department of Mechanical Engineering, Tokyo City University, Tokyo 158-8557, Japan; g2081010@tcu.ac.jp (Y.O.); g2181032@tcu.ac.jp (R.T.); tfujima@tcu.ac.jp (T.F.)

**Keywords:** PEDOT, organic thermoelectric materials, thermoelectric properties, first-principle calculation

## Abstract

PEDOT:Tos, a PSS-free PEDOT-based material, is a promising possible organic thermoelectric material for a practical conversion module because the material reportedly has a large power factor. However, since PEDOT:Tos is mainly reported to be a p-type thermoelectric material, the development of PSS-free PEDOT with n-type thermoelectric properties is desirable. Thus, in order to search for PSS-free PEDOT with n-type thermoelectric properties, we investigated the doping concentration of PTSA dependence of the thermoelectric property using the first-principle calculation. The band structure and the density of state indicated that the n-type thermal electromotive force was attributed to the electrons’ large effective mass. Such electrons were produced thanks to the binding of the dopant PTSA to the benzene ring. The contribution of the electron to the Seebeck coefficient increased with increasing PTSA doping concentrations.

## 1. Introduction

The demands for energy harvesting technologies are increasing with the expanding use of Internet of Things (IoT) devices [1]. Additionally, thermoelectric conversion is expected as the power source for wearable devices [2,3] and self-powered wireless devices [4] because thermoelectric conversion can generate electricity from waste heat and body heat. The thermoelectric materials used as the main parts in thermoelectric conversion modules are evaluated by the dimensionless figure of merit *ZT* = *S*^2^*σT*/*κ*, where *S*, *σ*, *T*, and *κ* are the Seebeck coefficient, electrical conductivity, temperature, and thermal conductivity [5]. *ZT* indicates that high-performance thermoelectric materials have a high power factor *PF* = *S*^2^*σ* and a small *κ*. A typical thermoelectric module is composed of p- and n-type thermoelectric conversion materials. In inorganic thermoelectric conversion materials, for thermal stress relief at each junction point in the module, the creation of p- and n-type in the same material system has been investigated [6,7].

Organic thermoelectric materials are better suited for energy harvesting devices than inorganic ones because of their smaller thermal conductivity [8,9,10], lower production cost, and higher bendability. Poly(3,4-ethylenedioxythiophene) doped with polystyrene sulphonic acid (PEDOT:PSS), one of the conductive polymers, is considered for practical use due to its high electrical conductivity and chemical stability. PEDOT without doping has only the π-conjugated conduction path and no conduction carriers; however, through doping with PSS, PEDOT can gain electrical conductivity and film formability [11]. PEDOT:PSS has been reported to have *ZT* = 0.42 [12], which is the highest value among organic thermoelectric materials. However, in order to be used as a stand-alone power source, an energy conversion efficiency of more than 10%, namely ZT ≥ 1, which is equivalent to other cogeneration systems, is required [13].

PEDOT:PSS has been reported to have a low thermal conductivity of 0.2 W/mK [9]. Subsequently, the reduction in thermal conductivity by orientation control has been studied [14], but a value below 0.2 W/mK has not been reported. Thus, the improvement of *ZT* by increasing the Seebeck coefficient and electrical conductivity is the main subject of investigation. To improve the Seebeck coefficient, the creation of a PEDOT:PSS composite with Cu_2_Se, an inorganic thermoelectric material, has been investigated, and *PF* = 389.7 μV/mK^2^ has been reported [15].

On the other hand, to improve electrical conductivity, the microstructure formed by PEDOT and PSS has been widely improved. It is reported that PEDOT doped with PSS forms a core-shell structure, in which a PEDOT core is covered by a PSS shell [16]. The formation of the core-shell structure is one factor that hinders the improvement of electrical conductivity [17] due to the insulator, PSS, interfering with the band conduction of PEDOT. An improvement in electrical conductivity has been reported for PEDOT:PSS, in which the addition of ethylene glycol removes excess PSS [18,19,20]. This measure has also been used to study the thermoelectric properties of PEDOT:PSS [21]. Since the formation of the core-shell structure is caused by PSS, PSS-free PEDOT-based conductive films have also been reported [22,23]. Furthermore, deposition on surface-modified substrates such as hierarchical nanoporous layered glasses [24] and polyelectrolyte brushes [25] has also been studied.

PEDOT doped with Tosylate (PEDOT:Tos) of PSS-free PEDOT has been reported to have *PF* = 453 μV/mK^2^, which is as large as PEDOT:PSS [26], and semi-metallic properties [27]. Thus, it is suggested that doping with small molecules improves the performance of PEDOT-based thermoelectric conversion materials. However, most of the thermoelectric properties of PSS-free PEDOT reported so far are p-type [26,27]; hence, the development of n-type materials is required. In this study, we focused on the doping of p-toluenesulfonic acid (PTSA) into PSS-free PEDOT. PTSA is used as an oxidizing agent for the polymerization of PEDOT:Tos, and it is known to exist in PEDOT as an anion, Tos. Therefore, the causal relationship between thermoelectric properties and doping concentration has only been investigated in models with the introduction of Tos [28,29], and the causal relationship with PTSA doping has not been investigated. Therefore, for the exploration of PSS-free PEDOT with n-type thermoelectric properties, we investigate the causal relationship between thermoelectric properties and the PTSA doping of PSS-free PEDOT using the first-principle band calculation.

## 2. Methods

**Figure 1:**
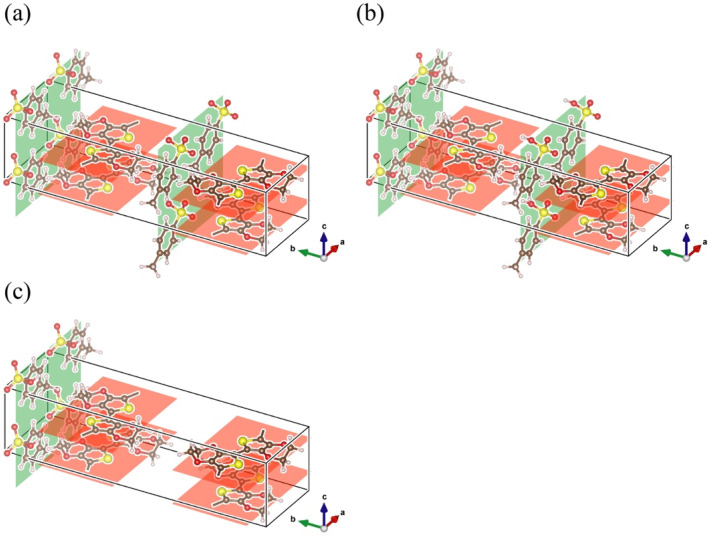
Calculation models of (**a**) PEDOT:Tos, (**b**) highly doped PEDOT:PTSA, and (**c**) poorly doped PEDOT: PTSA. The red, yellow, dark brown, and gray spheres indicate the atoms of oxygen, sulfur, carbon, and hydrogen. The red and green areas indicate PEDOT and dopant layer.

To calculate the band structure and the density of state (DOS), a first-principle band calculation based on the full potential augmented plane wave + local orbitals (APW + lo) method with a generalized gradient approximation with Perdew-Burke-Ernzerhof parametrization (GGA-PBE) was performed by using a WIEN2k package [30]. We calculated, using the models of PEDOT:Tos, highly doped PEDOT doped with PTSA (PEDOT:PTSA) and poorly doped PEDOT:PTSA shown in Figure 1a–c. Figure 1, based on the X-ray diffraction of PEDOT:Tos, was reported by K. E. Aasmundtveit et al. [31] and was drawn using VESTA [32]. In a self-consistent cycle, k-mesh was 7 × 2 × 8, and the cut-off energy was 290 eV.

## 3. Results and Discussion

### 3.1. Band Structure

**Figure 2:**
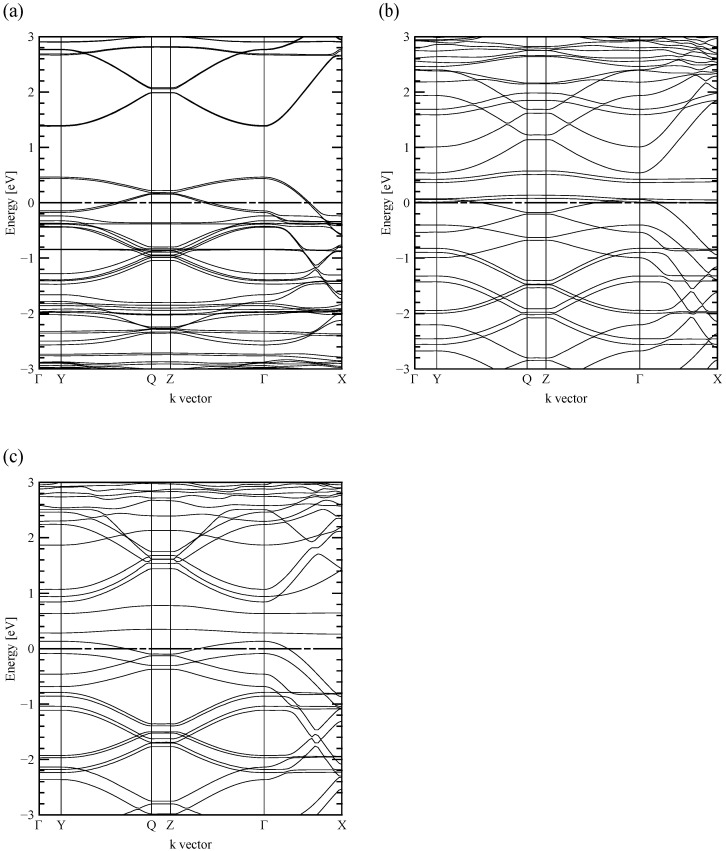
Band structure of (**a**) PEDOT:Tos, (**b**) highly doped PEDOT:PTSA, and (**c**) poorly doped PEDOT:PTSA. The Fermi energy is at 0 eV. The high-symmetry k-points in the first Brillouin zone are Γ = (0, 0, 0), Y = (0, 0.5, 0), Q = (0, 0.5, 0.5), Z = (0, 0, 0.5), and X = (0.5, 0, 0).

Figure 2a–c show the band structures of PEDOT:Tos, highly doped PEDOT:PTSA, and poorly doped PEDOT:PTSA. The Fermi level *E*_F_ was set to 0 eV, and the high symmetry k-points in the first Brillouin zone are Γ = (0, 0, 0), Y = (0, 0.5, 0), Q = (0, 0.5, 0.5), Z = (0, 0, 0.5), and X = (0.5, 0, 0).

In all models, the ΓY direction consisted only of the flat bands. The curvature of the k-vector dependence of the energy level is proportionate to the electron effective mass’ reciprocal [33]; thus, the larger the band’s curvature, the higher the electrical conductivity. The flat band, such as the ΓY direction in Figure 2, indicated infinite effective mass, namely a localized electron state. Since the ΓY direction showed electrical conduction between PEDOT through dopant molecules in real space, electrons are bound and localized in PEDOT or dopants. The localized electrons may exhibit insulator-like behavior or hopping conduction depending on the constraint’s strength.

In the ΓX and ΓZ directions corresponding to the a- and c-axis directions in Figure 1, the bands around the EF that contribute to electrical conduction in PEDOT:Tos and poorly doped PEDOT:PTSA consisted only of curved bands. In contrast, the ΓX and ΓZ structure of the highly doped PEDOT:PTSA consisted of flat bands located slightly higher than the EF in addition to the curved bands as the poorly doped PEDOT:PTSA. In all the models, the band’s curvature in the ΓX direction—the PEDOT chains’ bonding direction—was more significant than that in the ΓZ direction, which indicates the direction between the PEDOT. The band conduction in the ΓY direction was blocked, suggesting that the PEDOT chain’s electrical conduction is dominant regardless of the dopant.

### 3.2. DOS

**Figure 3:**
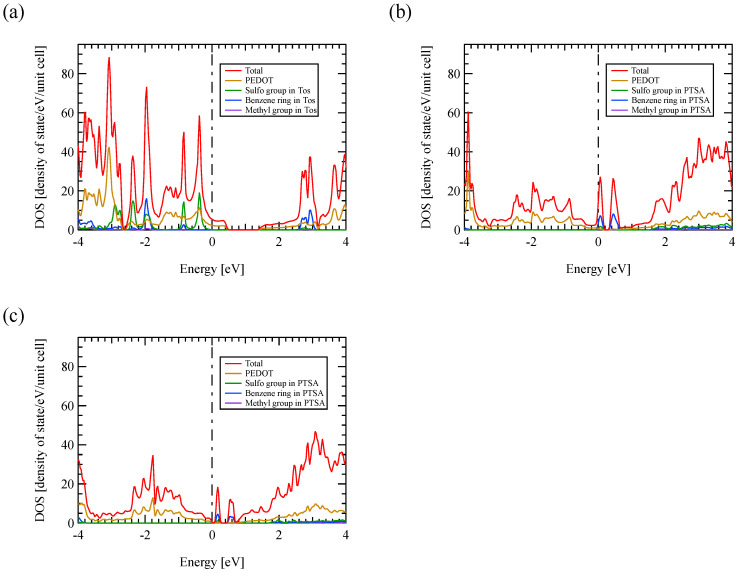
TDOS and PDOS projected for PEDOT and sulfo group, benzene ring, and methyl groups in the dopant of (**a**) PEDOT:Tos, (**b**) highly doped PEDOT:PTSA, and (**c**) poorly doped PEDOT:PTSA. The Fermi energy is at 0 eV.

The total density of state (TDOS) and the projected density of state (PDOS) of PEDOT:Tos, highly doped PEDOT:PTSA, and poorly doped PEDOT:PTSA are shown in Figure 3a–c, with a Fermi level *E*_F_ of 0 eV. The dopant forms the discrete density of states (DOS) in all models, whereas PEDOT forms the continuous DOS. The PDOS for PEDOT does not have a similar shape to the discrete DOS due to the dopant, suggesting weak orbital hybridization between PEDOT and the dopant. Thus, the covalent bonding between PEDOT and the dopant should be weak. 

The Seebeck coefficient depends on the difference in carriers’ contribution to electrical conduction at energies of 1.5 *k*_B_*T* and −1.5 *k*_B_*T* [34], where *k*_B_ is the Boltzmann’s constant. In discussions of the Seebeck coefficient of PEDOT-based thermoelectric conversion materials, approximations based on Mott’s theory are often used [27,28]. This paper also discusses this using Equation (1), one of the approximations based on Mott’s theory [35]:

(1) $$S\left(T\right)=-\frac{\pi^{2}k_{B}{}^{2}T}{3eD\left(E_{F}\right)}{\left[\frac{\partial D\left(\varepsilon \right)}{\partial \varepsilon }\right]}_{\varepsilon =E_{F}}$$

where *ε*, *D*(*ε*), and *e* represent the energy, the DOS at *ε*, and the charge of the carrier, respectively.

${\left[\partial D\left(\varepsilon \right)/\partial \varepsilon \right]}_{\varepsilon =E_{F}}$ calculated from Figure 3 for each model is shown in Table 1. The negative gradient of TDOS at the Fermi level for PEDOT:Tos and poorly doped PEDOT:PTSA indicated their positive Seebeck coefficients, whereas highly doped PEDOT:PTSA did the opposite, indicating a negative Seebeck coefficient. Thus, the thermoelectric properties changed from p-type to n-type with increasing PTSA doping concentrations. The highly doped PEDOT:PTSA had a ten-fold larger gradient than the other two models. The doping of PTSA possibly enhances the Seebeck coefficient because the PTSA-doped PEDOT exhibited a larger TDOS gradient than the Tos-doped one.

The positive TDOS gradient for the highly doped PEDOT:PTSA is supposed to come from the significant impurity level just above the valence-band edge caused by the PTSA’s benzene ring, as Figure 3b shows. The impurity level corresponds to the flat bands around the *E*_F_ in Figure 2b, and the states’ high density was attributed to the large effective mass’ localized electrons.

The electrons with large effective mass are considered to hardly contribute to the electrical conduction. The carriers should contribute to the conduction by their large mobility to develop the thermoelectric force. However, Equation (1) is approximated by the assumption that the carriers have sufficient mobility, and the Seebeck coefficient’s magnitude is discussed only by ${\left[\partial D\left(\varepsilon \right)/\partial \varepsilon \right]}_{\varepsilon =E_{F}}$. To obtain high n-type thermoelectric properties, the carriers in this impurity level must contribute to the electrical conduction by hopping conduction or the phonon drag. 

Contrastingly, Figure 2a and Figure 3a showed that PEDOT:Tos’ impurity levels differ from highly doped PEDOT:PTSA but several sharp peaks appear in the valence band from −3 to 0 eV, which are caused by a flat band. The peak at −0.4 eV was attributed to the sulfo group, while the other peaks were attributed to the benzene ring and sulfo group, similar to the highly doped PEDOT:PTSA’s impurity level. Tos’ structure has a hydrogen atom missing from the tip of the sulfo group of PTSA, suggesting that the band is formed only by the sulfo group due to this atomic defect. 

Conversely, Figure 2c and Figure 3c showed that the impurity level in the highly doped PEDOT:PTSA is also in the poorly doped PEDOT:PTSA. This impurity level was located at 0.17 eV in the bandgap for the poorly doped PEDOT:PTSA. Therefore, it should not contribute to the Seebeck coefficient and the electrical conductivity in the temperature range below 350 K, a practical usage temperature of organic thermoelectric materials. 

The impurity level was located within the bandgap and at the valence band edge for the poorly doped and highly doped PEDOT:PTSA. A lower impurity level indicates that the carriers are more easily excited from the valence band. As the valence band is attributed to the PEDOT molecule and the impurity level to the benzene ring and sulfo group of PTSA, the conductive carriers in PEDOT:PTSA are considered to be generated by PTSA pulling out electrons from PEDOT. The increase in the number of PTSA near the PEDOT by doping at higher concentrations is thought to attract more electrons in the PEDOT and weaken the electron binding in the PEDOT. This suggests that the impurity level shifts from within the bandgap to the valence band edge as the PTSA doping concentration increases.

To obtain n-type thermoelectric properties, the PTSA must attract electrons from the PEDOT; hence, more PTSA are required to be close to the PEDOT. As shown in the calculation model in this paper, if PTSA can be inserted between the oriented PEDOT chains, n-type PEDOT can be developed more efficiently. The orientation of PEDOT molecular chains can also contribute to the improvement of electrical conductivity. Therefore, by doping PTSA into the orientation-controlled PEDOT, it is possible to fabricate n-type PEDOT with higher thermoelectric properties.

## 4. Conclusions

We investigated the doping effect of PTSA on the thermoelectric properties of PSS-free PEDOT by first-principle calculation in order to search for n-type PSS-free PEDOT. The calculated band structure and the density of state revealed that the PTSA doped in PEDOT formed an energy band with a large effective mass in the bandgap. Furthermore, the impurity level originating from this band was shown to degenerate and overlap with the valence band edge with a high doping of PTSA. As the impurity band is sharp and intense, the p-n control of PEDOT:PTSA by its doping concentration is indicated to be possible.

## Figures and Tables

**Table 1:** ${\left[\partial D\left(\varepsilon \right)/\partial \varepsilon \right]}_{\varepsilon =E_{F}}$ calculated from TDOS shown in Figure 3.

Model	${\left[\partial D\left(\varepsilon \right)/\partial \varepsilon \right]}_{\varepsilon =E_{F}}$
PEDOT:Tos	−10.50
Highly doped PEDOT:PTSA	285.22
Poorly doped PEDOT:PTSA	−19.63
